# ﻿*Yushaniarubrovaginata* (Poaceae, Bambusoideae), a new combination for Sasarubrovaginata

**DOI:** 10.3897/phytokeys.255.147252

**Published:** 2025-04-24

**Authors:** Xing Li, Jing-Bo Ni, Mian Liang, Yi-Hua Tong, Nian-He Xia

**Affiliations:** 1 State Key Laboratory of Plant Diversity and Specialty Crops/Key Laboratory of Digital Botanical Garden of Guangdong Province, South China Botanical Garden, Chinese Academy of Sciences, Guangzhou 510650, China; 2 South China National Botanical Garden, Chinese Academy of Sciences, Guangzhou 510650, China; 3 Key Laboratory of State Forestry Administration on Tropical Forestry Research, Research Institute of Tropical Forestry, Chinese Academy of Forestry, Guangzhou 510520, China; 4 The Administration Center of Guangxi Cenwanglaoshan National Nature Reserve, Baise 530000, China

**Keywords:** Bamboo, Cenwanlaoshan Mountain, *Sasa*, taxonomy, *Yushania*

## Abstract

*Sasarubrovaginata* is transferred to *Yushania* based on morphological and molecular evidence. The lectotype for *S.rubrovaginata* is designated. Besides, a revised description of this species and a key to the four *Yushania* species distributed in Cenwanlaoshan Mountain are also provided.

## ﻿Introduction

*Sasa* Makino & Shibata (1901) is a medium genus of Arundinarieae, Bambusoideae (Zhang et al. 2020a), and characterized by having a shrubby habit, leptomorph rhizomes, single branch per culm node, spikelets arranged into a panicle-like inflorescence, and six stamens and three stigmas per floret (Hu 1996; Wang and Stapleton 2006; Clayton et al. 2016; Yi et al. 2008; Shi et al. 2022). About 43 species are recognized in *Sasa* genus at present, which are mainly distributed in East Asia (Vorontsova et al. 2016; Soreng et al. 2022; Li et al. 2023a; POWO 2025). Previous phylogenetic studies indicated that *Sasa* is polyphyletic (Peng et al. 2008; Sungkaew et al. 2009; Triplett and Clark 2010; Zeng et al. 2010; Zhang et al. 2012; Qin et al. 2021), and most Chinese *Sasa* species were successively revised and transferred to *Sinosasa* L. C. Chia ex N. H. Xia et al., *Yushania* Keng f., or *Pseudosasa* Makino ex Nakai in recent studies (Qin et al. 2021; Li et al. 2022, 2023a, 2023b, 2023c). For now, there are only three accepted names under *Sasa* from China (POWO 2025), viz., *S.subglabra*McClure (1940), *S.hainanensis* C. D. Chu & C. S. Chao (Chao et al. 1980) and *S.rubrovaginata* C. H. Hu (1985).

**Figure 1.**
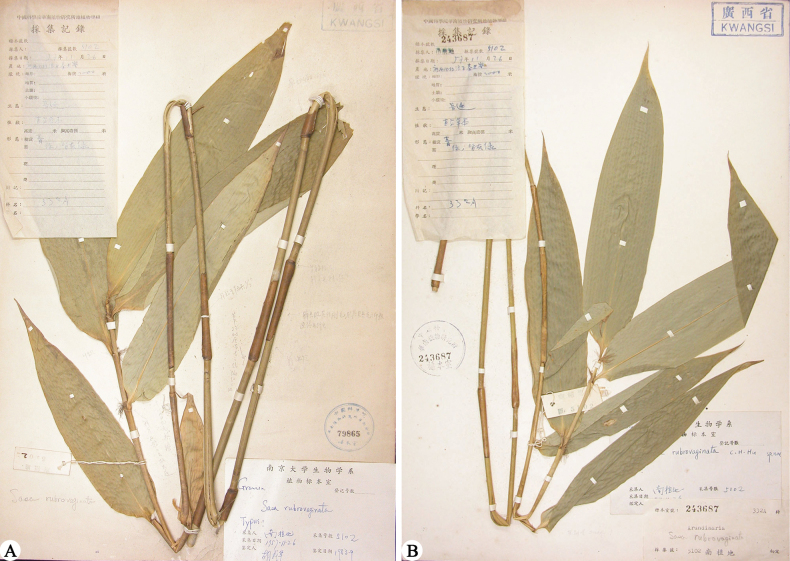
Lectotype (**A**) and isolectotype (**B**) of *Sasarubrovaginata* C. H. Hu (*Nanzhi 5102*, N **A** N019023168 **B** N019023159). Photos downloaded from Chinese Virtual Herbarium (https://www.cvh.ac.cn/).

*Sasarubrovaginata* was described based on the only collection, *Nanzhidi* (Phytogeography expeditions of South China Institute of Botany) 5102, from Cenwanglaoshan Mountain in Guangxi, China. Only two duplicates of the type were found in N (Fig. 1). Both duplicates (N019023159 and N019023168) only constitute a leafy branch. No other specimens can be located in the Chinese herbaria. Without any information of rhizome and branch complement, which are very important for the placement of generic position, it was designated as a member of *Sasa*. Thus, in order to ascertain the taxonomic position of *S.rubrovaginata*, it is necessary to recollect complete specimens with rhizome and branch complement from the type locality.

## ﻿Materials and methods

The complete specimens of *Sasarubrovaginata* were collected from the type locality, i.e., Cenwanlaoshan Mountain, Langping Town, Tianlin County, Guangxi Zhuang Autonomous Region, China, during a field trip in September 2022. Fresh leaf samples were stored in sealed bags with silica gel for DNA extraction. Magnifier and ruler (0.5 mm scale) were used for observations and measurements. Some minor characters were observed with a stereo microscope (Mshot-MZ101, Guangzhou Micro-shot Technology Co., Ltd, Guangzhou, China). Photos of type specimens of *S.rubrovaginata* were downloaded from the Chinese Virtual Herbarium (https://www.cvh.ac.cn/) for comparison. The description was conducted based on both living and dried materials, and the descriptive terms follow McClure (1966) and Beentje (2016). Herbarium acronyms follow Thiers (2025).

To study the phylogenetic position of *S.rubrovaginata* within the tribe Arundinarieae, phylogenetic analyses were carried out by using the complete chloroplast genome data. A total of 30 representatives from all the five subtribes of the tribe Arundinarieae (Zhang et al. 2020a) were sampled with *Dendrocalamusstrictus* (Roxburgh) Nees from the tribe Bambuseae as an outgroup. All the sampled taxa, information of voucher specimens and GenBank accession numbers were listed in Table 1.

**Table 1.** A list of the vouchers and GenBank accession numbers for the sampled species in this study.

Taxon	Voucher information	Accession number
**Ingroup**		
*Acidosasaglauca* B.M.Yang	CZY56 (IBSC)	OP850353
*Ampelocalamusactinotrichus* (Merr. & Chun) S.L.Chen, T.H.Wen & G.Y.Sheng	MPF10003 (KUN)	MF066245
*Chimonobambusatumidissinoda* Ohrnb.	MPF10083 (KUN)	MF066244
*Fargesiaalbocerea* J.R.Xue & T.P.Yi	ZhangYu-QuD588 (SANU)	NC_043891
*Fargesiacommunis* T.P.Yi	ZhangYu-QuD540 (SANU)	NC_043934
*Fargesiadaminiu* T.P.Yi & J.Y.Shi	ZhangYu-QuG660 (SANU)	NC_043942
*Fargesiahygrophila* J.R.Xue & T.P.Yi	ZhangYu-QuD552 (SANU)	NC_043938
*Fargesiasetosa* T.P.Yi	ZhangYu-QuG695 (SANU)	NC_043939
*Gaoligongshaniamegalothyrsa* (Hand.-Mazz.) D.Z.Li, Hsueh & N.H.Xia	MPF10056 (KUN)	JX513419
*Gelidocalamusstellatus* T.H.Wen	BH102 (IBSC)	OP850347
*Hsuehochloacalcareus* (C.D.Chu & C.S.Chao) D.Z.Li & Y.X.Zhang	MPF10050 (KUN)	KJ496369
*Indocalamuslongiauritus* Hand.-Mazz.	MPF10168 (KUN)	HQ337795
*Indocalamussinicus* (Hance) Nakai	ZMY037 (KUN)	MF066250
*Indosasacrassiflora* McClure	BH58 (IBSC)	OK558536
*Oligostachyumsulcatum* Z.P.Wang & G.H.Ye	Not provided by the author	MW190089
*Phyllostachysedulis* (Carriere) J.Houzeau	MPF10163 (KUN)	HQ337796
*Pleioblastusmaculatus* (McClure) C.D.Chu & C.S.Chao	CZY56 (IBSC)	JX513424
*Pseudosasacantorii* (Munro) Keng f.	MPF10006 (KUN)	MF066255
*Pseudosasajaponica* (Siebold & Zucc. ex Steud.) Makino ex Nakai	Pjc-1 (ZJFC)	KT428377
*Ravenochloawilsonii* (Rendle) D.Z.Li & Y.X.Zhang	MPF10146 (KUN)	JX513421
*Sasarubrovaginata* C.H.Hu	LX178 (IBSC)	PQ010623
*Sasaveitchii* (Carriere) Rehder	LC1325 (ISC)	KU569975
*Shibataeachiangshanensis* T.H.Wen	ZLN-2011080 (KUN)	MF066257
*Sinosasafanjingshanensis* N.H.Xia, Q.M.Qin & J.B.Ni	BH124 (IBSC)	OP850348
*Sinosasalongiligulata* (McClure) N.H.Xia, Q.M.Qin & J.B.Ni	CZY163 (IBSC)	OP850351
*Sinobambusatootsik* (Makino) Makino ex Nakai	NH031 (IBSC)	OP850357
*Yushaniaconfusa* (McClure) Z.P.Wang & G.H.Ye	ZhangYu-QuF642 (SANU)	NC_043893
*Yushaniamaculata* T.P.Yi	Not provided by the author	OR750784
*Yushanianiitakayamensis* (Hayata) Keng f.	Not provided by the author	MN310560
*Yushaniashuichengensis* T.P.Yi & L.Yang	Not provided by the author	OR750781
**Outgroup**		
*Dendrocalamusstrictus* (Roxburgh) Nees	zmy018 (KUN)	MK679802

### ﻿DNA extraction, sequencing, assembly and annotation

Total genomic DNA was extracted from dried leaves using the modified CTAB method (Li et al. 2013) and sent to Novo Gene Company (Beijing, China) for DNA assessment. The genomic DNA was then cut up to 350 bp-sized fragments for the construction of the library, and paired-end sequencing was performed on the Illumina Hiseq 4000 platform. A total of 20 G clean data (150 bp read length) were generated from each sample. These clean data were utilized to assemble the plastome by GetOrganelle v.1.7.7 pipeline (Jin et al. 2020) using the plastome of *Phyllostachysedulis* (Carriere) J. Houzeau (accession number: HQ337796) as the reference, with k-mer values of 45, 65, 85, 105, 125, word size of 102, and extension rounds of 20. Bandage software (Wick et al. 2015) was used to visually check if the final result of the assembled genome was circular or not. Finally, the assembled plastome sequence with the same direction as the reference sequence was kept and manually operated in Geneious v. 9.1.4 (Kearse et al. 2012) with the structure of LSC-IRa-SSC-IRb.

### ﻿Phylogenetic analysis

By using MAFFT v. 7.490, all of the complete chloroplast genomes were concatenated into a data matrix after being aligned. Maximum Likelihood (ML) and Bayesian Inference (BI) tools in the PhyloSuite v.1.2.3 platform (Xiang et al. 2023; Zhang et al. 2020b) were utilized for phylogenetic reconstructions. The Bayesian Information Criterion (BIC) in ModelFinder (Kalyaanamoorthy et al. 2017) was used to identify the optimal substitution model for ML and BI methods. Maximum likelihood phylogenies were inferred by using IQ-TREE v 2.2.0 (Nguyen et al. 2015) under the best-fit K81u+R4+F model. We then performed 1000 ultrafast bootstrap replicates and 1000 approximate likelihood ratio (SH-aLRT) tests to assess branch supports (Guindon et al. 2010). Bayesian Inference phylogenies were inferred using MrBayes v 3.2.7 (Ronquist et al. 2012) under the GTR+I+G+F model (2 parallel runs, 40,000,000 generations), in which the initial 25% of sampled data were discarded as burn-in. ML and BI trees were visualized in FigTree v. 1.4.4 (http://tree.bio.ed.ac.uk/software/figtree/).

## ﻿Results

### ﻿The real identity of *Sasarubrovaginata*

**Figure 2.**
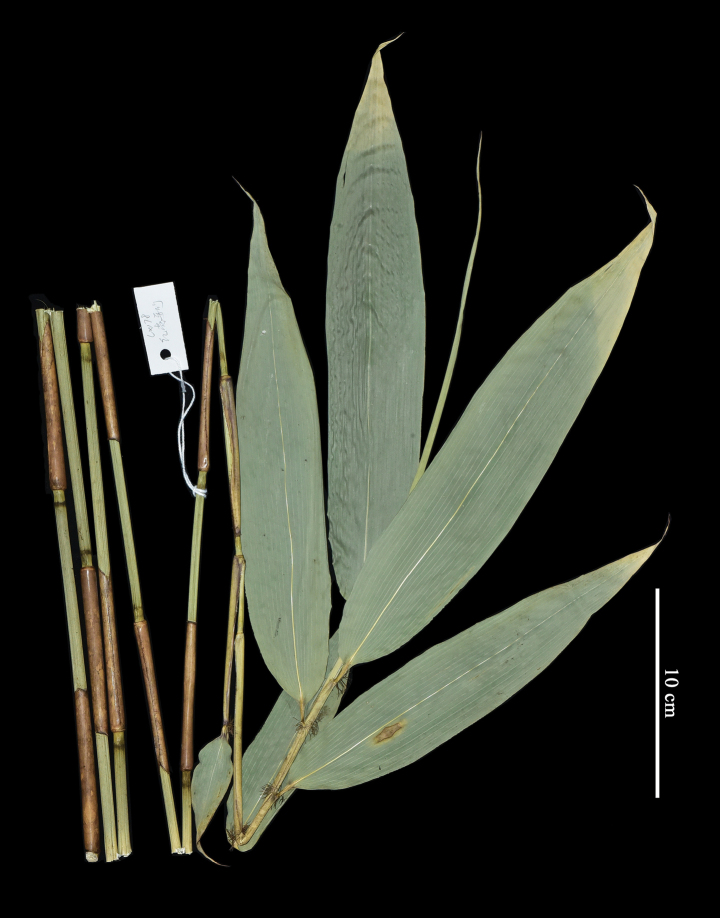
A specimen of *Sasarubrovaginata* C. H. Hu collected from the type locality, *X.Li & J.B.Ni LX178* (IBSC). Photo by Xing Li.

**Figure 3.**
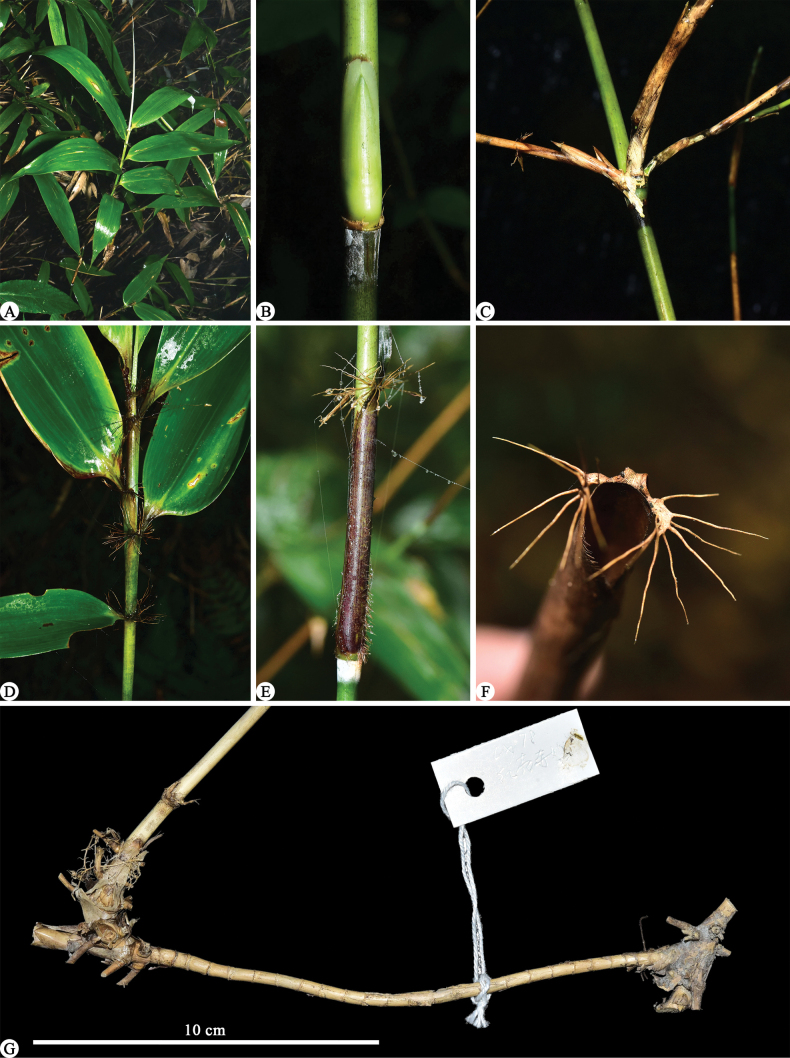
*Yushaniarubrovaginata***A** foliage leafy branch **B** culm bud **C** three branches at an upper culm node **D** partial foliage leafy branch, showing auricles and oral setae **E** culm leaf and white powdery infranodal region **F** apex of culm leaf sheath, showing ligules, auricles and oral setae **G** pachymorph rhizome with long neck. All photos by Xing Li.

According to the protologue and field note of the type specimens of *Sasarubrovaginata*, the gathering was collected at an elevation of 2000 m at Cenwangling (岑王岭) (the main peak of Cenwanlaoshan), Langping (浪平), Tianlin County (田林县). During our field trip to Cenwanlaoshan Mountain, only one bamboo species that has a long-necked pachymorph rhizome and two or three branches on the mid or upper culm nodes were found at the elevation from 1968 m to 2062.5 m (the peak’s elevation). The young and unbranched culms with foliage leaves at the apex (Fig. 2) fully matched with the protologue and the type specimens (Fig. 1) of *S.rubrovaginata* as well as the glabrous internodes, white powdery infranodal region, purple-red culm leaf sheath with a hispid base and ciliate margins, caducous culm leaf blades, truncate culm leaf ligules, 5–6 foliage leaves per ultimate branch with the glabrous sheath being ciliate on margins, falcate foliage leaf auricles with developed oral setae, truncate foliage leaf inner ligules, and glabrous foliage leaf blades with conspicuous transverse veins and 5–6 pairs secondary veins. Thus, we are very sure that the specimens we collected are *S.rubrovaginata*. Hu (1985) should take the young and unbranched culm with foliage leaves at the apex as a leafy branch, and supposed that the bamboo possessed solitary branch complement and leptomorph rhizome, just like the case of *S.tomentosa* C. D. Chu & C. S. Chao (≡ *Yushaniatomentosa* (C. D. Chao & C. S. Chao) N. H. Xia et al.; see Li et al. 2023a for nomenclatural revision). Owing to the long-necked rhizome (Fig. 3G) and branch complement with mostly solitary branch at lower culm nodes and two to three (Fig. 3C) branches at mid and upper culm nodes, it should be a member of *Yushania* rather than *Sasa*.

After examining the type specimens and referring to the related literature (Yi et al. 2008; Shi et al. 2022; Li et al. 2023a), *S.rubrovaginata* is mostly similar to *Y.tomentosa* by sharing the branch complement with usually solitary branch at lower culm nodes and two to three branches at mid and upper culm nodes (Fig. 3C), the glabrous internodes with purple spots, the culm leaf with sheath being ciliate on margins and leaving persistent remains on internode when falling off, truncate ligule, falcate auricles with radiate oral setae (Fig. 3F), and foliage leaf with sheath being ciliate on margins, falcate auricles with radiate oral setae (Fig. 3D), truncate ligule and glabrous blades, but differs in having purple-red (vs. green to brown) and densely brown hispid (Fig. 3E) (vs. white to yellowish-brown hirsute) culm leaf sheath, and glabrous culm leaf ligules margin (vs. white ciliolate), foliage leaf sheath (vs. densely white hirsute), outer ligule margin (vs. white ciliate) and pseudopetiole (vs. white puberulous). A more detailed comparison of the two species is presented in Table 2.

**Table 2.** Comparison of *Sasarubrovaginata* and *Yushaniatomentosa*.

Characters	*Sasarubrovaginata*	*Yushaniatomentosa*
**Culm leaf**		
**Sheath**	Purple-red when fresh, densely brown hispid	Green to brown when fresh, densely white to yellowish-brown hirsute
**Ligule**	Glabrous	White ciliolate on the margin
**Foliage leaf**		
**Sheath**	Glabrous, initially white powdery	Densely white hirsute
**Pseudopetiole**	Glabrous	White puberulous
**Outer ligule**	Glabrous	White ciliate on the margin

### ﻿Phylogenetic analysis

**Figure 4.**
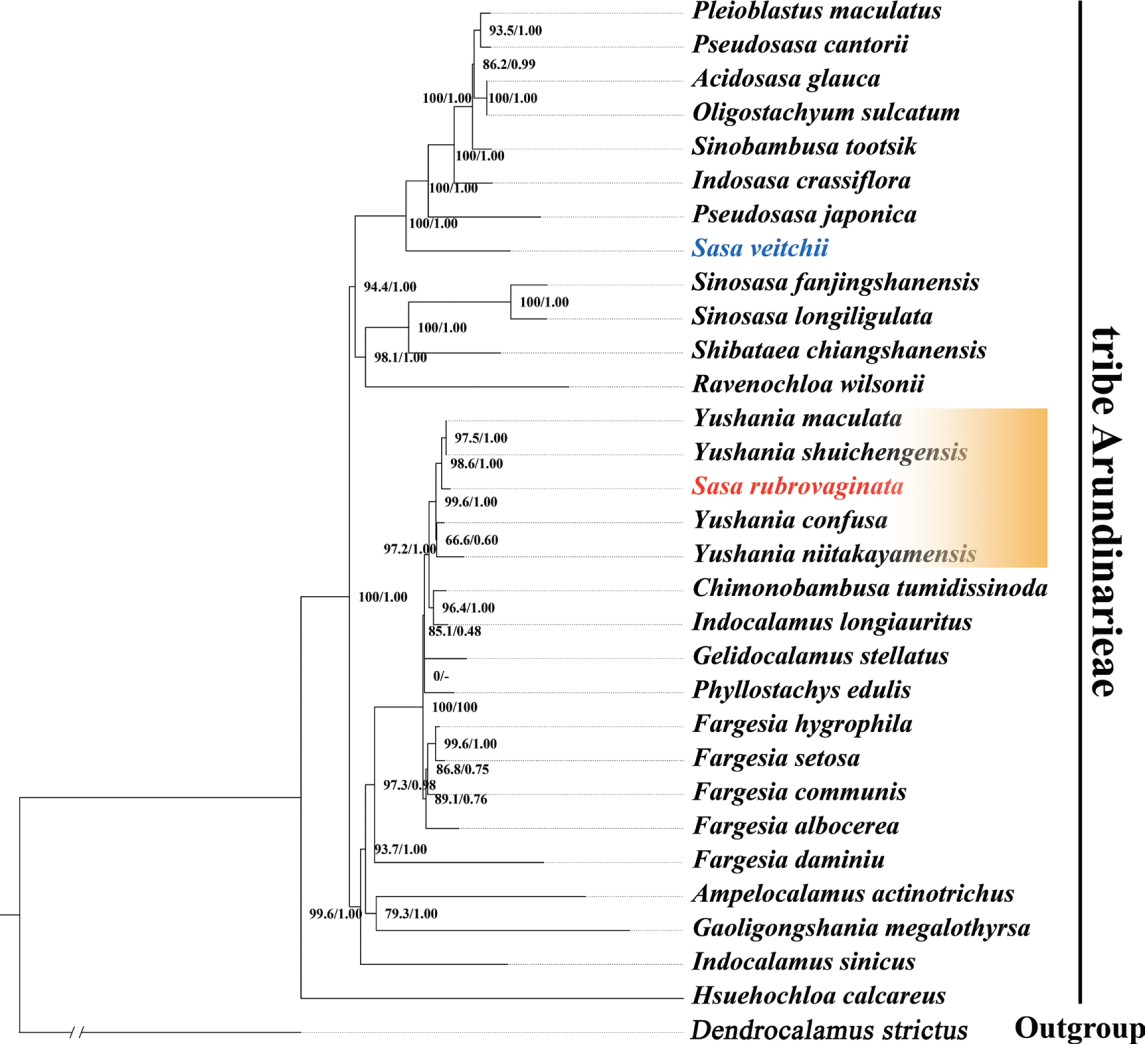
Phylogenetic tree derived from MI and BI methods based on the complete chloroplast sequences, showing the phylogenetic position of *Sasarubrovaginata*. Bootstrap values and posterior probabilities are indicated at each node, and the type of *Sasa* is highlighted in blue.

The chloroplast genomes of the sampled species vary from 139,404 bp (*Dendrocalamusstrictus*) to 140,064 bp (*Gaoligongshaniamegalothyrsa* (Hand.-Mazz.) D. Z. Li, Hsueh & N. H. Xia) with an alignment of 144,047 bp. Sequence divergence was observed in this data matrix with 3,903 variable sites (2.71%) comprising 3,087 singleton variable sites (2.14%) and 816 parsimony informative sites (0.57%). Only the ML tree was displayed (Fig. 4) with nodal support values from both ML and BI methods labeled on each node. As shown in the phylogenetic tree, *S.rubrovaginata* is distantly related to *S.veitchii* (Carriere) Rehder (= *S.albomarginata* (Miq.) Makino & Shibata, the type of *Sasa*) but forms a monophyletic clade with four *Yushania* species with strong nodal support (BS = 99.6% & PP = 1.00), which also supports that *S.rubrovaginata* should be a member of *Yushania*, rather than *Sasa*.

## ﻿Discussion

Based on the morphological and molecular evidence, it is concluded that *Sasarubrovaginata* represents a distinct species of *Yushania*. Accordingly, a new combination of *S.rubrovaginata* under *Yushania* should be made. Hu (1985) designated the collection *Nanzhidi 5102* (N) as the type of *S.rubrovaginata*, but there are two duplicates in N, which should be recognized as syntypes. We designated N019023168 (Fig. 1A) which bears some pencil annotations in Chinese and “Typus” in the identification slip by the author and has relatively better developed culm and foliage leaves as the lectotype of *S.rubrovaginata*.

### ﻿Taxonomic treatment

#### ﻿ Yushania rubrovaginata

Taxon classificationPlantaePoalesPoaceae

(C. H. Hu) N. H. Xia, Y. H. Tong, J. B. Ni & X. Li comb. nov.

715041CD-46A7-5E7E-8B28-0BF54BFE6B83

urn:lsid:ipni.org:names:77360579-1

Figs 1 , 2 , 3

##### Basionym.

*Sasarubrovaginata* C. H. Hu, Bamboo Research 2(2): 59 (1985)

##### Type.

China. • Guangxi: Tianlin County, Langping Town, Cenwanlaoshan Mountain, elev. 2000 m, 26 Nov. 1957, *Nanzhidi 5102* (lectotype N019023168!, Fig. 1A, designated here; isolectotype N019023159!, Fig. 1B)

##### Description.

Shrubby bamboo. Rhizomes pachymorph, necks 15–30 cm long, 3–5 mm in diameter, solid. Culms 1–3.5 m tall, 5–8 mm in diameter, diffuse; branches usually solitary at lower culm nodes, 2–3 at mid and upper culm nodes; internodes terete, 10–30 cm long, glabrous, densely purple-spotted, thickly white powdery below nodes, hollow; supranodal ridges raised. Culm buds solitary, long-ovate, yellow to light green, ciliate on the margin. Culm leaf sheaths persistent or tardily deciduous, ca. 1/2 as long as internodes, purple-red, densely brown hispid abaxially, densely ciliate on the margin; sheath scar prominent, with persistent remains of sheath base; auricles falcate, 3–5 × 1–2 mm; oral setae developed, radiate; ligule truncate, ca. 0.5 mm high; blades linear-lanceolate to lanceolate, reflexed, easily deciduous, margin serrulate. Foliage leaves 5–13 per ultimate branch; sheath glabrous, margin ciliate; auricles falcate, 1–3 × 0.5–1 mm; oral setae radiate, ca. 1 cm long; inner ligule truncate, ca. 1 mm high; outer ligule and pseudopetioles glabrous; blades broad-lanceolate to lanceolate, 17–26 × 3.5–6 cm, wavy when dry, glabrous, apex acuminate, base cuneate to obtuse; secondary veins 9–10 pairs, transverse veins conspicuous. Inflorescence unknown.

##### Distribution and habitat.

It is only found in Cenwang Mountain, Tianlin County, Guangxi, China. It grows on top of mountains at an altitude of 1968 to 2062.5 meters (the peak’s elevation).

##### Phenology.

New shoots from August to September.

##### Chinese name.

红壳玉山竹 (Chinese pronunciation: hóng ké yù shān zhú).

##### Notes.

Hu (1985) described that the culm leaf auricles and oral setae of *Yushaniarubrovaginata* are undeveloped (see Fig. 1). In fact, this bamboo does have developed culm leaf auricles and oral setae (Fig. 3E, F). But the culm leaf auricles and oral setae are easy to fall off, which exactly happens to the type specimens of *Y.rubrovaginata*. Similarly, the culm leaf sheaths of the type specimens are glabrous on the mid and upper parts, and only the basal part is sparsely hispid. Actually, *Y.rubrovaginata* has a thoroughly hispid culm leaf sheath, and pits are left after the trichomes fall off (Fig. 3E).

Wang and Stapleton (2006) treated *Sasaduplicata* W. T. Lin & Z. J. Feng (1992) as a synonym of *Y.rubrovaginata*. However, the former has a leptomorph rhizome, rather than pachymorph rhizome. Actually, *S.duplicata* owns some characters that are the same as *Pseudosasacantorii* (Munro) P. C. Keng ex S. L. Chen et al. (Zhu et al. 2006), such as glabrous internodes, the white powdery infranodal region, branch complement with one to three branches at each culm node, culm leaf sheath with ciliate margin, arcuate to truncate culm leaf ligules and falcate auricles with developed oral setae, foliage leaf sheath with ciliate margin, truncate ligules, developed oral setae and lanceolate to oblong-lanceolate blades with conspicuous transverse veins. Thus, *S.duplicata* is probably a synonym of *P.cantorii*, but more work needs to be done to ascertain this.

There are another three *Yushania* species, viz., *Y.cartilaginea* T. H. Wen (1984), *Y.chingii* T. P. Yi and *Y.rugosa* T. P. Yi (1986), distributed in Cenwanglaoshan Mountain. *Yushaniarubrovaginata* can be distinguishable from them by having branch complement with the solitary branch at lower culm nodes and two to three branches at mid and upper culm nodes, densely purple-spotted internodes, purple-red and densely brown hispid culm leaf sheath, and foliage leaf with developed and radiate oral setae. A key to these *Yushania* species is provided as follows.

### ﻿Key to *Yushania* species distributed in Cenwanglaoshan Mountain

**Table:** 

1a	Branches solitary at each culm node	**2**
2a	Culm leaf sheath glabrous; auricles developed and falcate; oral setae radiate with trichomes 5–8 mm long	***Y.chingii***
2b	Culm leaf sheath glabrous or sparsely purple-brown setose at base; auricles absent or small; oral setae absent or weak with trichomes 2–3 mm long	***Y.rugosa***
1b	Branches 1–3 at each culm node	**3**
3a	Internodes densely purple-spotted; culm leaf sheath purple-red, densely brown hispid; foliage leaf sheath with radiate oral setae	***Y.rubrovaginata***
3b	Internodes green, without purple spots; culm leaf sheath green to brown, glabrous; foliage leaf sheath with straight oral setae	***Y.cartilaginea***

### ﻿Additional specimens examined

*Yushaniarubrovaginata*: China. • Guangxi: Tianlin County, Langping Town, Cenwanglaoshan Mountain, Cenwangling, 25 September 2022, 24°29'22.4"N, 106°24'5.3"E, elev. 2062 m, *X. Li & J. B. Ni LX178* (IBSC).

*Yushaniatomentosa*: China. • Guangxi: Rongshui County, Jiuwan Mountain, elev. 1400 m, 25 August 1958, *S. H. Chun 15320* (isotypes: NAS00070361, image; WUK0211330, image; N019023167, image; IFP15899999w0005, image); • Rongshui County, Wangdong Township, Jiuwan Mountain, Weilinjiang, 23 September 2022, 25°18'39.3"N, 108°38'13.2"E, elev. 1358 m, *X. Li & J. B. Ni LX168* (IBSC).

*Yushaniacartilaginea*: China. • Guangxi: Baise City, [Tianlin County], Kashan [Laoshan = Cenwanlaoshan] forestry station, elev. 1700 m, 9 April 1982, *W. W. Chou L82433* (holotype: ZJFI).

*Yushaniachingii*: China. • Guangxi: Tianlin County, Laoshan forestry station, elev. 1400 m, 14 January 1990, *J. P. Ruan 90005* (N 019025132, image; N 019025139, image; N 019025140, image).

*Yushaniarugosa*: China. • Guizhou: Wangmo County, Maoping community, elev. 1500–1556 m, 26 August 1981, *T. P. Yi 81118* (holotype: SIFS).

## Supplementary Material

XML Treatment for Yushania rubrovaginata

## ﻿Additional information

### Author ORCIDs

Xing Li https://orcid.org/0000-0001-7146-0125

Yi-Hua Tong https://orcid.org/0000-0002-5034-005X

Nian-He Xia https://orcid.org/0000-0001-9852-7393

### Data availability

All of the data that support the findings of this study are available in the main text.
